# Efficacy of Whole-Blood Exchange Transfusion in Refractory Severe Autoimmune Haemolytic Anaemia Secondary to Systemic Lupus Erythematosus: A Real-World Observational Retrospective Study

**DOI:** 10.3389/fimmu.2022.861719

**Published:** 2022-06-10

**Authors:** Ying Jiang, Hong Jun Zhao, Hui Luo, Bi Juan Li, Zhi Min Zhang, Li Dan Zhao, Xiao Xia Zuo

**Affiliations:** ^1^Department of Rheumatology, Xiangya Hospital, Central South University, Changsha, China; ^2^Department of Blood Transfusion, Xiangya Hospital, Central South University, Changsha, China; ^3^Department of Rheumatology and Clinical Immunology, Peking Union Medical College Hospital, Chinese Academy of Medical Sciences and Peking Union Medical College, Beijing, China

**Keywords:** whole-blood exchange transfusion, autoimmune haemolytic anaemia, systemic lupus erythematosus, intravenous immunoglobulin, glucocorticoid

## Abstract

**Background:**

Severe autoimmune haemolytic anaemia (AIHA) in systemic lupus erythematosus (SLE) patients could be life-threatening and formidable, especially in those nonresponsive to glucocorticoids (GCs) and immunosuppressants (ISAs). Whole-blood exchange transfusion (WBE), with plasma exchange and pathogenic cell removal as well as healthy red blood cell transfusion, could be beneficial. The objective of this study was to investigate the efficacy and safety of WBE in combination with GCs/ISAs.

**Methods:**

In this retrospective study, the clinical data of 22 refractory severe SLE-AIHA inpatients between February 2016 and February 2021 were collected and analysed, among whom 14 patients had received WBE and were compared with those treated with typical second-line therapy of intravenous immunoglobulin and/or rituximab (IVIG/RTX).

**Results:**

Among the 22 severe refractory SLE-AIHA patients, eight patients received IVIG and/or RTX without WBE (group 1, IVIG/RTX, *n* = 8), seven patients were given WBE without IVIG/RTX (group 2, WBE alone, *n* = 7), and seven patients who failed initial IVIG/RTX therapy were given sequential WBE therapy (group 3 IVIG/RTX→WBE, *n* = 7). Fourteen patients had accepted WBE treatment regardless of prior IVIG/RTX usage (group 2 + 3, WBE ± IVIG/RTX, *n* = 14). On days 1, 3, 5, and 7 after corresponding therapies, patients of groups 2, 3, and 2 + 3 showed significantly higher levels of haemoglobin (Hb) than patients of group 1. Compared with patients of group 1, patients of groups 2, 3, and 2 + 3 took less time to reach and maintain Hb ≥60 g/L from baseline. Groups 2 and 2 + 3 consumed a lower dose of GCs than group 1 to reach and maintain Hb ≥60 g/L from baseline. Group 1 experienced longer hospital stays than group 2, and group 3’s cost of hospitalisation is more than groups 1 and 2. Hb_min_ <40 g/L may be a key indicative factor for initiating WBE remedy therapy as IVIG/RTX may not be effective enough in 48–72 h in those patients with refractory severe SLE-AIHA. No severe adverse effects were observed in the WBE group.

**Conclusions:**

WBE could be a safe and beneficial alternative therapy for refractory severe SLE-AIHA.

## Introduction

Autoimmune haemolytic anaemia (AIHA) is quite common in patients with systemic lupus erythematosus (SLE), and the incidence is approximately 5%–10% (1). Clinically, SLE-AIHA can range from mild/quiescent haemolysis to brisk and life-threatening conditions. Severe anaemia caused by SLE-AIHA may lead to tissue hypoxia, aggravate organ dysfunction, and result in haemodynamic instability, which could be a fatal medical emergency (2).

Glucocorticoids (GCs) and immunosuppressants (ISAs) are the first-line therapies to stop acute haemolysis in SLE-AIHA. For severe SLE-AIHA, high-dose or even pulse GC is warranted, but about 10%–25% of patients may respond poorly (3, 4). Second-line therapy, including rituximab (RTX), intravenous immunoglobulin (IVIG), and therapeutic plasmapheresis (TPE), is usually considered in severe refractory patients (5). RTX, functioning *via* depletion of B cells—the autoantibody producers, is effective in some refractory SLE-AIHA. However, the effect of RTX is not promptly presented, and risks of infections are of concern (6). Meanwhile, supportive red blood cell (RBC) transfusion is often urgently needed for severely anaemic patients (Hb <60 g/L) to restore basic blood/oxygen supply in order to prevent vital malfunction (7, 8). However, due to the presence of large amounts of circulatory autoantibodies and activation of the complement system, RBC transfusion could be formidable and inefficacious in SLE patients (9). IVIG and TPE may help to improve RBC transfusion efficiency. IVIG, by neutralising pathogenic autoantibodies responsible for haemolysis, may improve shortened erythrocyte survival in severe AIHA with an effective rate of around 30%–40% (10, 11). TPE, capable of removing autoantibodies and other pathogenic and proinflammatory components temporarily, can alleviate the acute haemolysis caused by incompatible RBC transfusion, but its efficacy in treating AIHA is controversial (12–14). Therefore, the management of refractory severe SLE-AIHA patients is extraordinarily challenging for clinicians.

Whole-blood exchange (WBE) is a new strategy that partially removes whole blood from patients and replaces it with the donor’s healthy RBCs and fresh frozen plasma (15). Different from TPE, WBE can physically remove not only plasma but also destroyed RBCs, sensitised RBCs, and activated lymphocytes, therefore being beneficial for reducing the source of autoantigens and the production of autoantibodies. Previous studies showed that WBE was effective in extremely severe haemolysis and a rapid clinical improvement could be achieved after WBE (15–19). Promisingly, WBE may become an alternative to current second-line therapy in refractory severe SLE-AIHA. Aiming to explore the efficacy and safety of this therapeutic strategy, we carried out this retrospective study in patients with severe refractory SLE-AIHA. By analysing the real-world clinical data and comparing the efficacy of WBE with the current second-line therapy of IVIG/RTX, we hope to provide a solid evidence for applying this new strategy to the formidable clinical conditions encountered in SLE-AIHA.

## Materials and Methods

### Patients

Patients admitted to Xiangya Hospital of Central South University between February 2016 and February 2021 who fulfilled the following inclusion criteria were recruited: (1) Aged 14–65 years old; (2) Diagnosed with SLE according to one of the following: the ACR (American College of Rheumatology) 1997, SLICC 2012, or ACR 2019 classification criteria for SLE; (3) Confirmed with severe AIHA secondary to SLE with Hb <60 g/L on admission; (4) Nonresponsive to high-dose GC, which is defined as having no increase of Hb or continuous Hb <60 g/L after methylprednisolone >2 mg/kg/day for more than 3 consecutive days; (5) WBE or second-line therapy IVIG and/or RTX were administered; (6) Ineffective blood transfusion is defined as an incomplete increase of Hb after RBC transfusion (an increase of Hb of less than 10 g/L after transfusion of 2 units of RBCs) or rapid decrease after transient elevation (lasting for no more than 1 day); and (7) Potent ISA (mycophenolate mofetil, cyclosporine, cyclophosphamide, or tacrolimus) had been adopted either for treating AIHA or for dealing with other systemic involvements of SLE. The exclusion criteria were as follows: (1) Haemolytic anaemia secondary to malignancies, infections, drugs, or diseases other than SLE; (2) Haemolytic anaemia is caused by anticardiolipin antibody syndrome, thrombotic microangiopathy, thrombotic thrombocytopenic purpura, and haemophagocytic syndrome; (3) Anaemia is purely caused by other conditions other than autoimmunity, such as nutritional anaemia, haemorrhagic anaemia, or thalassemia; and (4) Pregnancy, trauma, and infection.

### Procedure of WBE

The apheresis device (COBE Spectra, Terumo BCT, Lakewood, CO, USA) was used in this process. The schematic diagram was presented in **Supplementary Appendix S1** with a detailed operational procedure recorded in the figure legend. The procedure complied with the instructions previously reported by Li et al. in our group (15). Briefly, disposable tubing of dual-needle TPE was installed on the machine with the access and return terminals connected to the patients’ bilateral veins and the middle pipeline connected to the collection bag for collecting patients’ whole blood and transfusion bags filled with donor’s packed RBCs and plasma, respectively. Inlet and outlet flow rates as well as the exchange volume were finely modified and calculated to maintain blood dynamic homeostasis and to guarantee sufficient replacement. Patients’ whole blood collected in the collection bag was discarded, and the equivalent total volume of the donor’s RBCs and plasma was alternately transfused. Antianaphylaxis drugs were preoperatively administered (for example, an intramuscular injection of 20 mg of promethazine). Sodium citrate was used as an anticoagulant. Calcium gluconate was given intravenously in case hypocalcaemia occurs. Generally, about 50%–80% of a patient’s total blood volume will be exchanged each time. All of the WBE-treated patients accepted this treatment only once.

### Statistical Analysis

The clinical data were presented as *n* (%) or mean ±SD. Continuous variables were compared using one-way analysis of variance (ANOVA) for comparisons among three groups with LSD employed as a *post-hoc* test for multiple comparisons. Independent-sample *t*-tests with or without Welch’s correction or paired-sample *t*-tests were used for comparisons between two groups, and Fisher’s exact test was employed for categorical parameter analysis. A Kaplan–Meier analysis was applied to demonstrate the improvement of haemolytic anaemia. The binary logistic regression analysis was used to identify the factors critical for beneficial outcomes, and the contingency table and Kruskal–Wallis ANOVA test were used for validation. All statistical analyses were conducted with GraphPad Prism 8.0 or SPSS 25.0 software packages. *p* < 0.05 was considered statistically significant. The choice of treatment was determined on the basis of clinicians’ and patients’ agreement, patients’ global disease condition, patients’ wishes, and coexisting medical conditions. Informed consent for the off-label use of WBE was obtained from all patients. The retrospective study protocol was approved by the ethics committees of Xiangya Hospital. Since this study was retrospective in nature, the requirement of informed consent was waived, and general confidential principles were obeyed.

## Results

A total of 22 severe refractory SLE-AIHA patients in our centre were included in this study. All of these patients had been prescribed GC+ISA as the basic standard of care (SOC). Among them, eight patients received IVIG and/or RTX without WBE (group 1, IVIG/RTX, *n* = 8), seven patients were given WBE without IVIG/RTX (group 2, WBE alone, *n* = 7), and seven patients who failed initial IVIG/RTX therapy were given sequential WBE therapy (group 3 IVIG/RTX→WBE, *n* = 7). Patients in group 3 who had suffered from severe clinical conditions of persistently decreasing Hb or unimproved clinical manifestations of hypoxia (fatigue, chest tightness, and rapid heart rate) or unstable vital signs 48–72 h after the start of the IVIG/RTX received WBE therapy immediately. In combination, 14 patients had accepted WBE treatment regardless of prior IVIG/RTX usage (group 2 + 3, WBE ± IVIG/RTX, *n* = 14).

**Figure 1:**
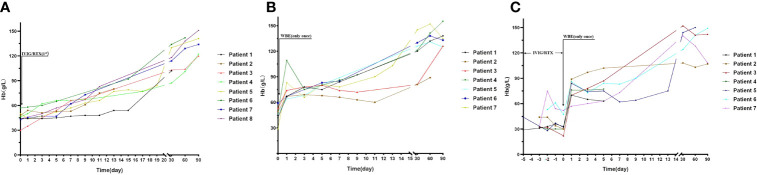
The alteration of Hb levels with time after second-line therapy (IVIG/RTX) and WBE in groups 1, 2, and 3. **(A)** Hb levels at different time points in patients of group 1. **(B)** Hb levels at different time points in patients of group 2. **(C)** Hb levels at different time points in patients of group 3. Group 1 (IVIG/RTX, *n* = 8); group 2 (WBE alone, *n* = 7); group 3 (IVIG/RTX→WBE, *n* = 7).

Demographic features and clinical parameters were comparable among the three groups (groups 1, 2, and 3), except for a higher positive rate of antinucleosome antibodies (anti-ANUA) antibody being observed in group 1 than that in group 2 (**Table 1**). There was also no significant difference in disease activity (SLEDAI score) and lupus-related organ involvement among the three groups (groups 1, 2, and 3) (**Table 1**). Comparisons of clinical features, systemic involvements, and laboratory indices between groups 1 and 2 + 3 also showed no difference (**Table 1**). The WBE procedure-related data of the 14 patients are shown in **Supplementary Table 1**. The alterations of Hb levels with time after IVIG/RTX or WBE treatment in groups 1, 2, and 3 are shown in **Figure 1**.

**Table 1:** Baseline clinical characteristics and treatment condition of patients from 3 groups.

	Group 1 (*n* = 8)	Group 2 (*n* = 7)	Group 3 (*n* = 7)	Group 2 + 3 (*n* = 14)	*F/p*	*p*			*p*
					Group 1 vs. 2 vs. 3	Group 1 vs. 2	Group 1 vs. 3	Group 2 vs. 3	Group 1 vs. 2 + 3
Age (years)	41.88 ± 15.20	36.43 ± 14.21	30.86 ± 13.92	33.64 ± 13.82	1.080/0.360	0.477	0.158	0.481	0.209
Female [*n* (%)]	7 (87.50%)	6 (85.71%)	6 (85.71%)	12 (85.71%)	–	1.000	1.000	1.000	1.000
SLE duration ≥1 year [*n* (%)]	5 (62.50%)	4 (57.14%)	4 (57.14%)	8 (57.14%)	–	1.000	1.000	1.000	1.000
AIHA duration (days)	18.50 ± 9.04	14.29 ± 10.18	18.43 ± 11.28	16.71 ± 10.40	0.405/0.673	0.432	0.989	0.454	0.635
Symptoms									
Fatigue or dizziness	8 (100%)	7 (100%)	7 (100%)	14 (100%)	–	1.000	1.000	1.000	1.000
Palpitation	6 (75.00%)	7 (100%)	7 (100%)	14 (100%)	–	0.467	0.467	1.000	0.121
Fever	2 (25.00%)	4 (57.14%)	4 (57.14%)	8 (57.14%)	–	0.315	0.315	1.000	0.204
Shortness of breath	2 (25.00%)	4 (57.14%)	2 (28.57%)	6 (42.86%)	–	0.315	1.000	0.592	0.649
Sclera jaundice	3 (37.50%)	3 (42.86%)	4 (57.14%)	7 (50.00%)	–	1.000	0.619	1.000	0.675
Dark urine	2 (25.00%)	3 (42.86%)	3 (42.86%)	6 (42.86%)	–	0.608	0.608	1.000	0.649
Petechiae or ecchymoses	1 (12.50%)	0 (0%)	2 (28.57%)	2 (14.28%)	–	1.000	0.569	0.462	1.000
Headache	1 (12.50%)	0 (0%)	1 (14.28%)	1 (7.14%)	–	1.000	1.000	1.000	1.000
Unconsciousness	0 (0%)	0 (0%)	1 (14.28%)	1 (7.14%)	–	1.000	0.467	1.000	1.000
Haematuria	1 (12.50%)	1 (14.28%)	0 (0%)	1 (7.14%)	–	1.000	1.000	1.000	1.000
Diarrhoea or vomiting	0 (0%)	1 (14.28%)	0 (0%)	1 (7.14%)	–	0.467	1.000	1.000	1.000
Arthritis	2 (25.00%)	1 (14.28%)	3 (42.86%)	4 (28.57%)	–	1.000	1.000	0.559	1.000
Rash	1 (12.50%)	2 (28.57%)	1 (14.28%)	3 (21.43%)	–	0.569	1.000	1.000	1.000
Oral ulcer	0 (0%)	1 (14.28%)	1 (14.28%)	2 (14.28%)	–	1.000	1.000	1.000	0.515
Edema	0 (0%)	2 (28.57%)	0 (0%)	2 (14.28%)	–	0.200	1.000	0.462	0.515
Splenomegaly	3 (37.50%)	5 (71.43%)	4 (57.14%)	9 (64.29%)	–	0.315	0.619	1.000	0.378
Indirect bilirubin (µmol/L)	21.95 ± 17.17	20.87 ± 18.56	38.74 ± 41.27	29.81 ± 32.11	0.948/0.405	0.940	0.252	0.239	0.531
Lactate dehydrogenase	584.88 ± 273.19	430.26 ± 201.49	497.90 ± 202.98	464.08 ± 197.45	0.847/0.444	0.211	0.476	0.590	0.244
Hb baseline (g/L)	45.25 ± 7.65	46.57 ± 9.43	38.14 ± 8.90	42.36 ± 9.83	1.951/0.170	0.771	0.128	0.084	0.483
Percentage of reticulocyte count (%)	16.45 ± 11.83	14.20 ± 11.62	22.69 ± 8.88	18.44 ± 10.87	1.145/0.339	0.695	0.283	0.162	0.692
Coombs test									
IgG+C3 [*n* (%)]	8 (100%)	7 (100%)	7 (100%)	14 (100%)	–	1.000	1.000	1.000	1.000
IgG [*n* (%)]	8 (100%)	7 (100%)	7 (100%)	14 (100%)	–	1.000	1.000	1.000	1.000
C3 [*n* (%)]	7 (87.5%)	6 (85.71%)	6 (85.71%)	12 (85.71%)	–	1.000	1.000	1.000	1.000
Indirect test [*n* (%)]	7 (87.5%)	6 (85.71%)	5 (71.43%)	11 (78.57%)	–	1.000	0.569	1.000	1.000
Other autoantibodies									
dsDNA [*n* (%)]	5 (62.5%)	5 (71.43%)	5 (71.43%)	10 (71.43%)	–	1.000	1.000	1.000	1.000
Sm [*n* (%)]	4 (50%)	4 (57.14%)	2 (28.57%)	6 (42.86%)	–	1.000	0.608	0.592	1.000
U1RNP [*n* (%)]	3 (37.50%)	4 (57.14%)	1 (14.28%)	5 (35.71%)	–	0.619	0.569	0.266	1.000
SSA [*n* (%)]	8 (100%)	4 (57.14%)	4 (57.14%)	8 (57.14%)	–	0.077	0.077	1.000	0.051
SSB [*n* (%)]	1 (12.50%)	4 (57.14%)	1 (14.28%)	5 (35.71%)	–	0.119	1.000	0.266	0.351
Ribosome P [*n* (%)]	2 (25.00%)	1 (14.28%)	1 (14.28%)	2 (14.28%)	–	1.000	1.000	1.000	0.602
ANUA [*n* (%)]	7 (87.50%)	1 (14.28%)	5 (71.43%)	6 (42.86%)	–	0.010	0.569	0.103	0.074
AHA [*n* (%)]	5 (62.50%)	2 (28.57%)	2 (28.57%)	4 (28.57%)	–	0.315	0.315	1.000	0.178
aPL [*n* (%)]	2 (25.00%)	2 (28.57%)	0 (0.00%)	2 (14.28%)	–	1.000	0.467	0.462	1.000
Immune globulin									
IgG (g/L)	20.80 ± 5.94	23.97 ± 10.96	20.81 ± 5.17	22.39 ± 8.39	0.403/0.674	0.436	0.997	0.453	0.642
IgA (mg/L)	2,757.50 ± 1,204.51	2,069.14 ± 536.95	2,870.00 ± 1,945.34	2,469.57 ± 1,432.61	0.732/0.494	0.337	0.874	0.281	0.637
IgM (mg/L)	2,101.88 ± 1,992.11	1,129.86 ± 460.12	1,392.29 ± 437.09	1,261.07 ± 452.14	–	0.218	0.356	0.295	0.276
Complement									
C3 (mg/L)	317.25 ± 113.53	371.57 ± 114.52	353.86 ± 178.43	362.71 ± 144.33	0.307/0.739	0.455	0.613	0.812	0.454
C4 (mg/L)	64.29 ± 32.77	57.73 ± 36.48	46.29 ± 37.46	52.01 ± 36.02	0.487/0.622	0.725	0.339	0.553	0.437
SLE related complications									
ITP [*n* (%)]	2 (25.00%)	4 (57.14%)	2 (28.57%)	6 (42.86%)	/	0.315	1.000	0.592	0.649
IL [*n* (%)]	4 (50%)	2 (28.57%)	0 (0.00%)	2 (14.28%)	/	0.608	0.077	0.462	0.161
LN [*n* (%)]	0 (0%)	2 (28.57%)	2 (28.57%)	4 (28.57%)	/	0.200	0.200	1.00	0.254
VTE [*n* (%)]	0 (0%)	0 (0%)	1 (14.28%)	1 (7.14%)	/	1.000	0.467	1.00	1.000
SLEDAI	7.63 ± 5.42	8.14 ± 3.44	7.00 ± 4.93	7.57 ± 4.13	0.103/0.903	0.834	0.801	0.655	0.979
GC therapy									
Glucocorticoid pulse [*n* (%)]	2 (25.0%)	2 (28.57%)	1 (14.28%)	3 (21.43%)	/	1.000	1.000	1.000	1.000
Total prednisone dose(mg) (from Hb baseline until Hb ≥60 g/L)	922.50 ± 511.63	185.71 ± 141.76	682.86 ± 271.89	434.29 ± 331.56	8.239/0.003	0.001	0.208	0.017	0.013
Second-line treatment									
IVIG [*n* (%)]	7 (87.5%)	/	7 (100%)	7 (50.00%)	/	/	1.000	/	0.167
RTX [*n* (%)]	1 (12.55)	/	1 (14.28%)	1 (7.14%)	/	/	1.000	/	1.000
Immunosuppressive agent									
Cyclosporin A [*n* (%)]	5 (62.50%)	2 (28.57%)	1 (14.28%)	3 (21.43%)	–	0.315	0.119	1.000	0.081
Tacrolimus [*n* (%)]	1 (12.50%)	1 (14.28%)	2 (28.57%)	3 (21.43%)	–	1.000	0.569	1.000	1.000
Cyclophosphamide [*n* (%)]	2 (25.00%)	5 (71.43%)	4 (57.14%)	9 (64.29%)	–	0.132	0.315	1.000	0.183
Treatment-related adverse reactions									
Infection [*n* (%)]	2 (25.0%)	0 (0%)	0 (0%)	0 (0%)	–	0.467	0.467	1.000	0.121
Anaphylaxis or others [*n* (%)]	0 (0.00%)	1 (14.28%)	1 (14.28%)	2 (14.28%)	–	0.467	0.467	1.000	0.515
Hospital stay (days)	18.00 ± 6.19	12.86 ± 2.04	14.00 ± 3.42	13.43 ± 2.77	–	0.055	0.154	0.462	0.080
Total cost of hospitalisation ($)	6,083.19 ± 1,515.38	4,013.82 ± 1,044.85	8,174.77 ± 2,285.60	6,094.24 ± 2,752.48	10.667/0.001	0.028	0.027	0.000	0.992

dsDNA, anti-dsDNA antibody; Sm, anti-Smith antibody; U1RNP, anti-U1RNP antibody; SSA, anti-SSA antibody; SSB, anti-SSB antibody; ribosome P, antiribosome P antibody; ANUA, antinucleosome antibody; AHA, antihistone antibody; aPL, antiphospholipid antibody including anticardiolipin antibody, anti-β2-glycoprotein 1, and lupus anticoagulant; ITP, immune thrombocytopenia; IL, immune leukopenia; LN, lupus nephritis; VTE, venous thrombosis; SLEDAI, systemic lupus erythematosus disease activity index. Group 1 (IVIG/RTX, n = 8); group 2 (WBE alone, n = 7); group 3 (IVIG/RTX→WBE, n = 7).

**Figure 2:**
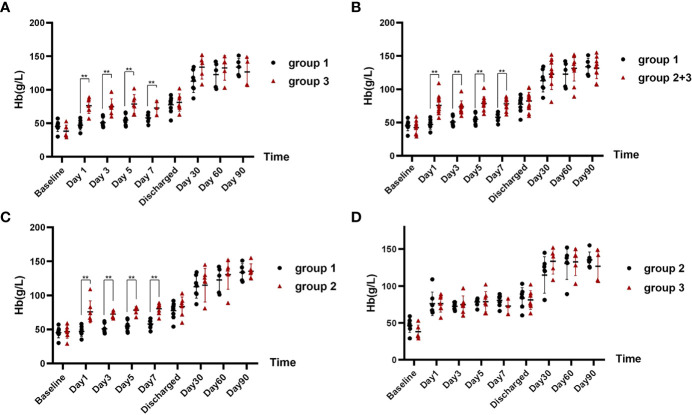
The comparison of Hb levels at different time points in each group after corresponding therapies. **(A)** Hb levels of patients in group 1 after IVIG/RTX treatment vs. that of patients in group 3 after WBE. **(B)** Hb levels of patients in group 1 after IVIG/RTX treatment vs. that of patients in group 2 + 3 after WBE. **(C)** Hb levels of patients in group 1 after IVIG/RTX treatment vs. that of patients in group 2 after WBE. **(D)** Hb levels of patients in group 2 after WBE treatment vs. that of patients in group 3 after WBE. Baseline is the time that IVIG/RTX begins in groups 1 and 3 and WBE therapy begins in group 2. Discharge is the time that patients are discharged from the hospital. Data are shown as the mean ± SD. Each dot plot represents an individual patient, ^**^p < 0.01. Group 1 (IVIG/RTX, *n* = 8); group 2 (WBE alone, *n* = 7); group 3 (IVIG/RTX→WBE, *n* = 7).

At baseline, the three groups were comparable on Hb levels (**Table 1**). On days 1, 3, 5, and 7 after corresponding therapies (IVIG/RTX for group 1 and WBE for groups 2 and 3), patients who had received WBE, regardless of prior IVIG/RTX administration or not (group 2 or 3 or 2 + 3), showed significantly higher levels of Hb than patients of group 1 (**Figures 2A–C**), though the preponderance faded with time and no difference could be observed at discharge and 1 month later. Additionally, there was no difference in Hb levels between patients in group 2 and those in group 3 after WBE therapy (**Figure 2D**). After the immediate acute phase, patients in all three groups continued GCs/ISAs for maintenance immunosuppressive therapy. Fifteen (68%) patients have been followed up to 6 months, 13 (59%) up to 9 months, and 11 (50%) to 12 months. The GC dosage was tapered to 7.5–10 mg prednisone (or equivalent)/day, and ISAs were maintained in most of the patients during the following 6 months. The Hb levels were comparable among groups 1, 2, and 3, and there was no difference in Hb levels between groups 1 and 2 + 3 either during the following 6–12 months after the immediate acute phase (**Supplementary Figure 1**). Two patients in group 1 had a mild relapse during 9–12 months of follow-up, and both patients maintained safe Hb levels by adjusting GC and/or ISA dosage.

**Figure 3:**
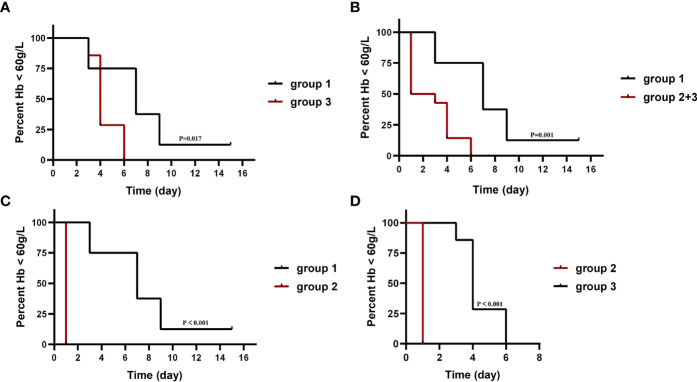
The median time taken from baseline to reach and maintain Hb ≥60 g/L in each group. The baseline is the Hb level when IVIG/RTX begins in groups 1 and 3 and WBE therapy begins in group 2. Group 1 (IVIG/RTX, *n* = 8); group 2 (WBE alone, *n* = 7); group 3 (IVIG/RTX→WBE, *n* = 7).

Compared with those not receiving WBE (patients of group 1), patients who received WBE, either alone (group 2) or followed IVIG/RTX as a remedy (group 3) or their collection (group 2 + 3, WBE ± IVIG/RTX) displayed the superiority in taking less time to reach and maintain Hb ≥60 g/L from baseline (group 2 vs. group 1, 1.0 vs. 7.5 days, *p* < 0.001; group 3 vs. group 1, 4.4 vs. 7.5 days, *p* = 0.017; group 2 + 3 vs. group 1, 2.7 vs. 7.5 days, *p* = 0.001) (**Figures 3A–C**). Furthermore, when looking into the total GC dose employed during the time from baseline to reaching Hb ≥60 g/L, both groups 2 and 2 + 3 had consumed a lower dose of GC than group 1 (group 2 vs. group 1, 185.71 ± 141.76 vs. 922.50 ± 511.63 mg, *p* = 0.001; group 2 + 3 vs. group 1, 434.29 ± 331.56 vs. 922.50 ± 511.63 mg, *p* = 0.013), whereas group 3 consumed equivalent GC dosage as group 1 (group 3 vs. group 1, 682.86 ± 271.89 vs. 922.50 ± 511.63 mg, *p* = 0.208) (**Table 1**). Surprisingly, though patients in group 3 had received IVIG/RTX prior to WBE therapy, they seemed to take a longer time to obtain Hb ≥60 g/L from baseline than patients of group 2 who received WBE alone (group 2 vs. group 3, 1.0 vs. 4.4 days, *p* < 0.001) (**Figure 3D**). In addition, patients in group 3 consumed more GCs than those in group 2 during the time from baseline to reaching Hb ≥60 g/L (group 2 vs. group 3, 185.71 ± 141.76 vs. 682.86 ± 271.89 mg, *p* = 0.017) (**Table 1**). The inferiority of IVIG/RTX plus WBE (group 3) to WBE alone (group 2) therapy may be explained by the ineffective second-line therapy of IVIG/RTX, which wasted time and necessitated longer or larger doses of GC usage.

Patients who accepted WBE (with prior IVIG/RTX or not) experienced shorter hospitalisation than patients who accepted traditional second-line therapy (IVIG/RTX) with an approximate statistical significance (group 2 + 3 vs. group 1, 13.43 ± 2.77 vs. 18.00 ± 6.19 days, *p* = 0.080), although the total cost of hospitalisation of the two groups was comparative (*p* = 0.992) (**Table 1**). Group 2 (WBE alone) experienced a shorter hospital stay (18.00 ± 6.19 and 12.86 ± 2.04 days for groups 1and 2, respectively) than group 1 (IVIG/RTX) with a marginal statistical significance (*p* = 0.055). Group 3’s (IVIG/RTX→WBE) cost of hospitalisation is more ($6,083.19 ± 1,515.38, $4,013.82 ± 1,044.85, and 8,174.77 ± 2,285.60 for groups 1, 2, and 3, respectively) than groups 1 and 2 (*p* = 0.027 and *p* < 0.001) **(** **Table 1****)**. Group 2 (WBE alone) was manifested with the lowest hospitalisation cost among the three, therefore displaying superiority over the other two groups.

**Figure 4:**
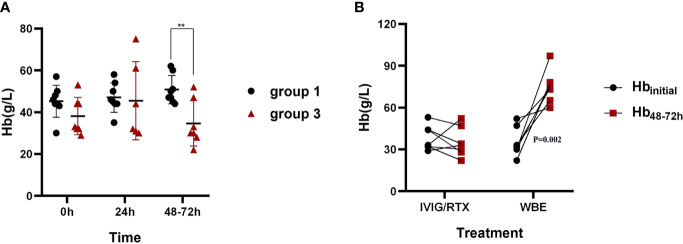
Hb levels in 48–72 h after corresponding therapies in groups 1 and 3. **(A)** The comparison of Hb levels in 48–72 h after IVIG/RTX treatment between groups 1 and 3. **(B)** The changes of Hb levels at 48–72 h after IVIG/RTX and WBE treatment of group 3. Hb_initial_ is the Hb level when IVIG/RTX or WBE therapy begins. Data are shown as the mean ± SD. Each dot plot represents an individual patient, ^**^p < 0.01. Group 1 (IVIG/RTX, *n* = 8), group 3 (IVIG/RTX→WBE, *n* = 7).

Group 3 showed no significant change in Hb levels at 48–72 h after IVIG/RTX treatment but displayed a distinct increase in Hb at 48–72 h after WBE treatment, which can be regarded as a kind of self-anterior-posterior contrast (**Figure 4**). To identify the necessity for WBE in those who already received IVIG/RTX, binary logistic regression analysis was performed with groups 1 and 3, and we found that Hb_min_ <40 g/L was a key indicative factor for initiating WBE remedy therapy as IVIG/RTX may not be effective enough in 48–72 h in those refractory severe SLE-AIHA patients (OR = 42.0; 95% CI, 2.136–825.715; *p* = 0.014) (**Table 2**). Validation *via* contingency table and Kruskal–Wallis ANOVA test confirmed this implication (Spearman value = 0.732; *p* = 0.002).

**Table 2:** The key factor for initiating WBE identified by binary logistic regression analysis.

Variables	OR	95% CI	*p*
Hb_min_ <40 g/L	42.0	2.136–825.715	0.014
Age >35 years	0.250	0.028–2.237	0.215
Total bilirubin elevated	2.222	0.280–17.631	0.450

Adverse reactions were observed in four cases of this cohort, among which two cases of nosocomial infection occurred in group 1 and two cases of WBE-related adverse reaction occurred in groups 2 and 3 with one in each. All adverse events were mild, and patients recovered rapidly. There were no serious adverse events (**Table 1**).

## Discussions

AIHA is triggered by the loss of tolerance to RBC autoantigens and aberrant activation of the complement system. Antibodies produced by autoreactive B cells (usually IgG) bind to RBC antigens, leading to the destruction of RBCs (8). SLE-AIHA is even more intractable and life-threatening than primary AIHA, with more diverse circulatory autoantibodies and more complicated systemic involvement (3). Despite aggressive GC and ISA therapy, as well as second-line therapies such as IVIG/RTX and TPE, some refractory severe SLE-AIHA patients show no improvements or respond slowly. To obtain rapid remission of life-threatening severe haemolysis (Hb <60 g/L), restore effective oxygen supplies, and avoid the aggravation of organ dysfunction due to severe anaemia, a new strategy is expected, and WBE may be a promising therapeutic way by providing the dual benefits of removing harmful components and supplementing fresh healthy RBCs.

In this small cohort retrieved from the past 5 years in our medical centre, WBE therapy based on SOC displayed advantages of quick recovery of Hb levels in refractory severe SLE-AIHA patients, which was superior to traditional IVIG/RTX treatment in shortening the duration of hypoxic conditions and protecting vital organs from the functional deterioration. Though this advantage is subdued with time and no difference in Hb levels can be observed 1 month later, the therapy with WBE still provides other benefits on shortening the time of hospitalisation, reducing the cost of hospitalisation, and tapering required GC dosage, which reduces the risk of nosocomial infection and medical consumption. The above facts imply that, for severe refractory SLE-AIHA patients, the advantage of WBE is embodied in medical emergency conditions, and the long-term benefits may still rely on GC+ISA therapy. We also found that Hb <40 g/L may act as an indicator for the determination of starting WBE other than second-line therapy. Switching on WBE in time may help restore necessary Hb levels as quickly as possible, shorten hospital stays, and reduce hospitalisation costs (15). Furthermore, our small cohorts showed WBE was safe, and only mild adverse reactions were reported.

Though this small cohort provided some evidence that WBE is effective for refractory severe SLE-AIHA, the mechanism is not fully clarified. It is postulated that the removal of sensitised and destroyed RBCs, activated white blood cells, and plasma will reduce the source of autoantigens, diminish autologous reactive lymphocytes, memory immune cells, and inflammatory mediators, and alleviate the damage of macrophages to RBCs in peripheral circulation (16).

There are several limitations in our study. First, the sample size is small and cases were collected in a single centre. These results need to be testified in a larger population from multicentres. Second, only a few patients chose RTX as the second-line treatment in this study which may cause an underestimation of its efficacy. Finally, due to the retrospective design of this study, data missing exists and bias is inevitable. To extrapolate the data to general clinical practice, further prospective studies with a larger sample size from multicentre are warranted. At present, our study provided some evidence that WBE as an effective add-on treatment based on GC+ISA, may be superior to current second-line therapy of IVIG/RTX in refractory severe SLE-AIHA, especially in those with a Hb lower than 40 g/L.

## Data Availability Statement

The raw data supporting the conclusions of this article will be made available by the authors, without undue reservation.

## Ethics Statement

The studies involving human participants were reviewed and approved by the ethics committees of Xiangya Hospital, Central South University. Written informed consent for participation was not required for this study in accordance with the national legislation and the institutional requirements.

## Author Contributions

YJ: substantial contributions to the conception and design of the work and the acquisition, analysis, and interpretation of data for the work; drafting the work and revising it critically for important intellectual content; and final approval of the version to be published. HZ: contributions to the acquisition, analysis, and interpretation of data for the work. HL: revising the work critically for important intellectual content and final approval of the version to be published. BL: contributions to the acquisition of data for the work and providing the procedure of WBE therapy and performing WBE therapy for the patients. ZZ: providing the procedure of WBE therapy and performing WBE therapy for the patients. LZ: contributions to the conception and design of the work and revising it critically for important intellectual content and final approval of the version to be published. XZ: contributions to the conception and design of the work and revising it critically for important intellectual content and final approval of the version to be published.

## Funding

This work was supported by Natural Science Foundation of Hunan Province 2021JJ40996.

## Conflict of Interest

The authors declare that the research was conducted in the absence of any commercial or financial relationships that could be construed as a potential conflict of interest.

## Publisher’s Note

All claims expressed in this article are solely those of the authors and do not necessarily represent those of their affiliated organizations, or those of the publisher, the editors and the reviewers. Any product that may be evaluated in this article, or claim that may be made by its manufacturer, is not guaranteed or endorsed by the publisher.

## Supplementary Material

Supplementary Table 1 WBE values in the procedure of the 14 patients in group 2 and group 3. Group 1 (IVIG/RTX, *n* = 8); group 2 (WBE alone, *n* = 7); group 3 (IVIG/RTX→WBE, *n* = 7).

Supplementary Figure 1 Hb levels of patients during follow-up 6-12 months after the immediate acute phase. **(A)** Hb levels during follow-up after the immediate acute phase in groups 1, 2, and 3. **(B)** Hb levels during follow-up after the immediate acute phase in groups 1 and 2 + 3. Data are shown as the Mean ± SD. Each dot plot represents an individual patient. NS No significance. Group 1 (IVIG/RTX, *n* = 8); group 2 (WBE alone, *n* = 7); group 3 (IVIG/RTX→WBE, *n* = 7).

Supplementary Appendix S1 The schematic of the whole blood exchange procedure. (AC, anticoagulant; *the returned RBCs line is clamped). The operational procedure of whole-blood exchange follows the following steps. Step 1: Switch on the power and install the disposable tubing of dual-needle TPE on the machine. Step 2: Infuse 0.9% normal saline in the pipeline and connect the two spikes with the bags of donor’s packed RBCs and plasma, respectively. Step 3: Input patient’s information (including sex, height, weight, and haematocrit), and the estimated total blood volume, centrifuge speed, anticoagulation ratio, plasma, and collected pump speed are calculated by the spectra system automatically. Step 4: Connect access and return terminals with bilateral veins. Step 5: Run the procedure and turn it into manual mode, and then clamp the returned RBC line (line b) so that the patient’s whole blood is removed to the collection bag from the removed plasma line (line a). Donor’s RBCs and plasma are sequentially transfused into the patient from the return plasma line (line c). Step 6: Adjust the inlet flow rate range from 10 to 40 ml/min according to the patient’s access pressure. In case of fluctuation of blood dynamic, the infusion rate of exchanged liquid is almost equal to the collection rate. The setting value is modulated slightly throughout the procedure according to the change in the patient’s condition. Step 7: Exit manual mode and turn off the power when the exchange program is finished. Antianaphylaxis drugs were preoperatively administered (For example, intramuscular injection of 20 mg promethazine). Sodium citrate was used as an anticoagulant. Calcium gluconate was given intravenously in case hypocalcaemia occurs. About 50%–80% of the patient’s total blood volume was exchanged each time. Plasma was preincubated at 37°C before transfusion, and a blood warmer was not strictly required throughout the whole procedure.
