# Supplementary material for: Efficacy of Whole-Blood Exchange Transfusion in Refractory Severe Autoimmune Haemolytic Anaemia Secondary to Systemic Lupus Erythematosus: A Real-World Observational Retrospective Study
Source: Front Immunol. 2022 Jun 10;13:861719. doi: 10.3389/fimmu.2022.861719 (PMC9226305; doi:10.3389/fimmu.2022.861719)
Supplement: Supplementary Table 1 — WBE values in the procedure of the 14 patients in group 2 and group 3. Group 1 (IVIG/RTX, n = 8); group 2 (WBE alone, n = 7); group 3 (IVIG/RTX→WBE, n = 7). [file Table_1.docx]

Supplementary Table S1 WBE values in the procedure of the 14 patients in group 2 and group 3

| Patient | Total blood volume (ml) | Inlet blood volume (ml) | RBCs (units) | Plasma  (ml) | Removed  (ml) | Exchange ratio  (%) | Time  (min) |
| --- | --- | --- | --- | --- | --- | --- | --- |
| Group 2-1 | 3611 | 2290 | 6 | 1050 | 2110 | 58.43 | 76 |
| Group 2-2 | 3402 | 1970 | 4.5 | 1000 | 1778 | 52.26 | 71 |
| Group 2-3 | 2961 | 2376 | 6 | 1000 | 1959 | 66.16 | 74 |
| Group 2-4 | 3699 | 2447 | 10 | 1000 | 2403 | 64.96 | 91 |
| Group 2-5 | 3463 | 2469 | 7 | 1000 | 2118 | 61.16 | 79 |
| Group 2-6 | 3578 | 2678 | 6 | 1000 | 2460 | 68.75 | 98 |
| Group 2-7 | 3386 | 2456 | 6 | 1000 | 2268 | 66.98 | 78 |
| Group 3-1 | 3046 | 2150 | 6 | 1000 | 1950 | 64.02 | 72 |
| Group 3-2 | 3278 | 2479 | 8 | 1000 | 2272 | 69.31 | 79 |
| Group 3-3 | 3678 | 2864 | 9.5 | 900 | 2642 | 71.83 | 104 |
| Group 3-4 | 3562 | 2438 | 6 | 900 | 2096 | 58.84 | 89 |
| Group 3-5 | 3872 | 2839 | 10 | 1000 | 2620 | 67.66 | 108 |
| Group 3-6 | 3169 | 2368 | 6 | 1000 | 1987 | 62.70 | 76 |
| Group 3-7 | 3758 | 2878 | 7 | 1000 | 2595 | 69.05 | 102 |
| Mean value | 3461.64±271.37 | 2478.71±1263.98 | 6.50±2.06 | 1010±22.36 | 2232.71±277.50 | 64.44±5.32 | 85.50±12.91 |

*Group 1 (IVIG/RTX, n=8); group 2 (WBE alone, n=7); group 3 (IVIG/RTX→WBE, n=7).
