# Supplementary figures and images for: Efficacy of Whole-Blood Exchange Transfusion in Refractory Severe Autoimmune Haemolytic Anaemia Secondary to Systemic Lupus Erythematosus: A Real-World Observational Retrospective Study
Source: Front Immunol. 2022 Jun 10;13:861719. doi: 10.3389/fimmu.2022.861719 (PMC9226305; doi:10.3389/fimmu.2022.861719)

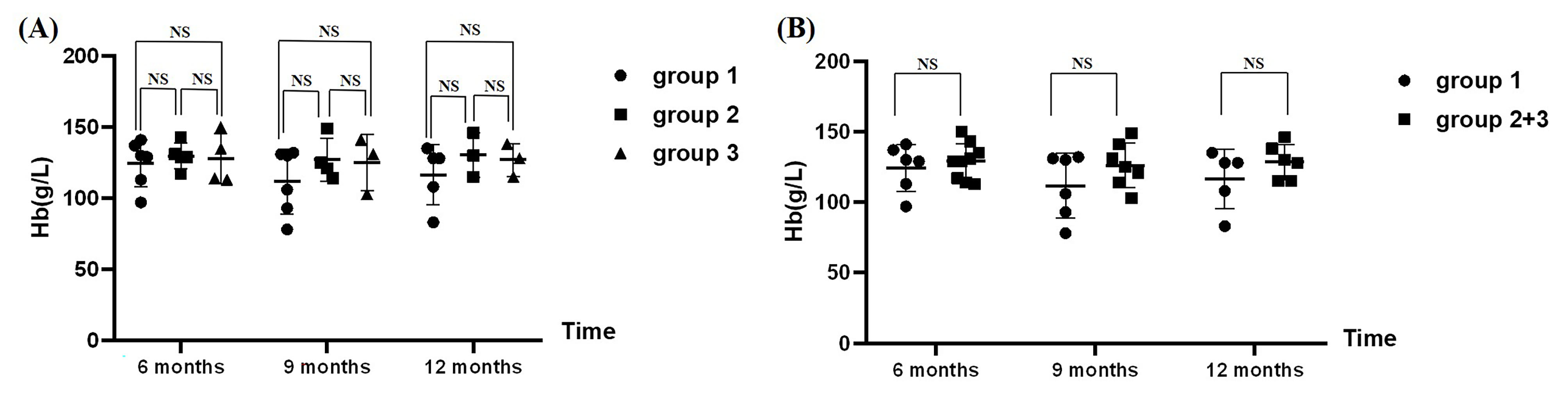

Supplement: Supplementary Figure 1 — Hb levels of patients during follow-up 6-12 months after the immediate acute phase. (A) Hb levels during follow-up after the immediate acute phase in groups 1, 2, and 3. (B) Hb levels during follow-up after the immediate acute phase in groups 1 and 2 + 3. Data are shown as the Mean ± SD. Each dot plot represents an individual patient. NS No significance. Group 1 (IVIG/RTX, n = 8); group 2 (WBE alone, n = 7); group 3 (IVIG/RTX→WBE, n = 7). [file Image_1.jpg]
