# Supplementary material for: Efficacy of Whole-Blood Exchange Transfusion in Refractory Severe Autoimmune Haemolytic Anaemia Secondary to Systemic Lupus Erythematosus: A Real-World Observational Retrospective Study
Source: Front Immunol. 2022 Jun 10;13:861719. doi: 10.3389/fimmu.2022.861719 (PMC9226305; doi:10.3389/fimmu.2022.861719)
Supplement: Supplementary Appendix S1 — The schematic of the whole blood exchange procedure. (AC, anticoagulant; *the returned RBCs line is clamped). The operational procedure of whole-blood exchange follows the following steps. Step 1: Switch on the power and install the disposable tubing of dual-needle TPE on the machine. Step 2: Infuse 0.9% normal saline in the pipeline and connect the two spikes with the bags of donor’s packed RBCs and plasma, respectively. Step 3: Input patient’s information (including sex, height, weight, and haematocrit), and the estimated total blood volume, centrifuge speed, anticoagulation ratio, plasma, and collected pump speed are calculated by the spectra system automatically. Step 4: Connect access and return terminals with bilateral veins. Step 5: Run the procedure and turn it into manual mode, and then clamp the returned RBC line (line b) so that the patient’s whole blood is removed to the collection bag from the removed plasma line (line a). Donor’s RBCs and plasma are sequentially transfused into the patient from the return plasma line (line c). Step 6: Adjust the inlet flow rate range from 10 to 40 ml/min according to the patient’s access pressure. In case of fluctuation of blood dynamic, the infusion rate of exchanged liquid is almost equal to the collection rate. The setting value is modulated slightly throughout the procedure according to the change in the patient’s condition. Step 7: Exit manual mode and turn off the power when the exchange program is finished. Antianaphylaxis drugs were preoperatively administered (For example, intramuscular injection of 20 mg promethazine). Sodium citrate was used as an anticoagulant. Calcium gluconate was given intravenously in case hypocalcaemia occurs. About 50%–80% of the patient’s total blood volume was exchanged each time. Plasma was preincubated at 37°C before transfusion, and a blood warmer was not strictly required throughout the whole procedure. [file DataSheet_1.docx]

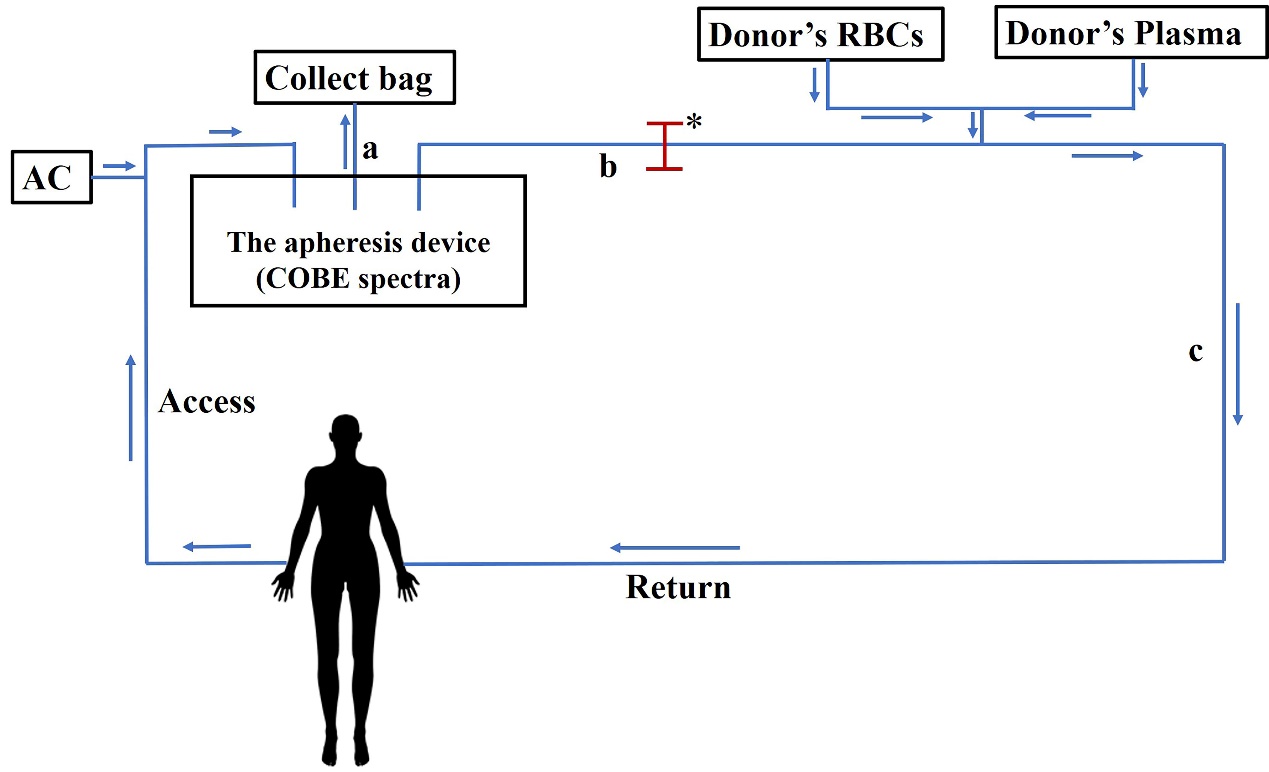


The schematic of the whole blood exchange procedure.

(AC: anticoagulant; *: the returned RBCs line is clamped.)

The operational procedure of whole-blood exchange follows the following steps. Step 1: Turn on the power and install the disposable tubing of dual-needle TPE on the machine. Step 2: Infuse 0.9% normal saline in the pipeline and connect the two spikes with the bags of donor’s packed RBCs and plasma, respectively. Step 3: Input patient’s information (including sex, height, weight and hematocrit), and the estimated total blood volume, centrifuge speed, anti-coagulation ratio, plasma and collected pump speed are calculated by the Spectra system automatically. Step 4: Connect access and return terminals with bilateral veins. Step 5: Run the procedure and turn into manual mode. And then clamp the returned RBCs line (line b) so that the patient’s whole blood is removed to the collect bag from removed plasma line (line a). Donor’s RBCs and plasma are sequential transfused into patient from the return plasma line (line c). Step 6: Adjust the inlet flow rate range from 10 to 40 ml/min according to the patient’s access pressure. In case of fluctuation of blood dynamic, infusion rate of exchanged liquid is almost equal to the collect rate. The setting value is modulated slightly throughout the procedure according to the change of patient’s condition. Step 7: Exit manual mode and turn off power when exchange program is finished (15).
